# Study protocol: a cluster randomized trial to evaluate the effectiveness and implementation of onsite GeneXpert testing at community health centers in Uganda (XPEL-TB)

**DOI:** 10.1186/s13012-020-00988-y

**Published:** 2020-04-21

**Authors:** Tania F. Reza, Talemwa Nalugwa, Katherine Farr, Mariam Nantale, Denis Oyuku, Annet Nakaweesa, Johnson Musinguzi, Moksha Vangala, Priya B. Shete, Austin Tucker, Olivia Ferguson, Katherine Fielding, Hojoon Sohn, David Dowdy, David A. J. Moore, J. Lucian Davis, Sara L. Ackerman, Margaret A. Handley, Achilles Katamba, Adithya Cattamanchi

**Affiliations:** 1 Division of Pulmonary and Critical Care Medicine, Zuckerberg San Francisco General Hospital, University of California San Francisco, San Francisco, CA USA; 2 Center for Tuberculosis, University of California San Francisco, San Francisco, CA USA; 3 Uganda Tuberculosis Implementation Research Consortium (U-TIRC), Kampala, Uganda; 4 Implementation Science Program, Johns Hopkins Bloomberg School of Public Health, Baltimore, MD USA; 5 Department of Epidemiology, Johns Hopkins Bloomberg School of Public Health, Baltimore, MD USA; 6 Faculty of Epidemiology and Population Health and TB Centre, London School of Hygiene and Tropical Medicine, London, UK; 7 Faculty of Infectious and Tropical Diseases and TB Centre, London School of Hygiene and Tropical Medicine, London, UK; 8 Department of Epidemiology of Microbial Diseases and Center for Methods in Implementation and Prevention Sciences, Yale School of Public Health, New Haven, CT USA; 9 Section of Pulmonary, Critical Care, and Sleep Medicine, Yale School of Medicine, New Haven, CT USA; 10 Department of Social and Behavioral Sciences, University of California San Francisco, San Francisco, CA USA; 11 Department of Epidemiology and Biostatistics, University of California San Francisco, San Francisco, CA USA; 12 School of Medicine, Makerere University College of Health Sciences, Kampala, Uganda

**Keywords:** Cluster randomized trial, Pragmatic trial, Effectiveness-implementation design, Tuberculosis, Xpert MTB/RIF

## Abstract

**Background:**

Delays in diagnosis and treatment of tuberculosis (TB) remain common in high-burden countries. To improve case detection, substantial investments have been made to scale-up Xpert MTB/RIF (Xpert), a cartridge-based nucleic acid amplification test that can detect TB within 2 hours, as a replacement for sputum smear microscopy. However, the optimal strategy for implementation of Xpert testing remains unclear.

**Methods:**

The Xpert Performance Evaluation for Linkage to Tuberculosis Care (XPEL-TB) trial uses an ultra-pragmatic, hybrid type II effectiveness-implementation design to assess the effectiveness and implementation of a streamlined strategy for delivery of Xpert testing in real-world settings. Twenty health centers with TB microscopy units were selected to participate in the trial, with ten health centers randomized to the intervention strategy (onsite molecular testing using GeneXpert Edge, process redesign to facilitate same-day TB diagnosis and treatment, and performance feedback) or routine care (onsite sputum smear microscopy plus referral of sputum samples to Xpert testing sites). The primary outcome is the number of patients with microbiologically confirmed TB who were initiated on treatment within 14 days of presentation to the health center, which reflects successful completion of the TB diagnostic evaluation process. Secondary outcomes include health outcomes (6-month vital status), as well as measures of the reach, adoption, and implementation of the intervention strategy.

**Discussion:**

The design elements and implementation approach for the XPEL-TB trial were intentionally selected to minimize disruptions to routine care procedures, with the goal of limiting their influence on key primary and secondary outcomes. Trial findings may result in increased support and funding for rapid, onsite molecular testing as the standard-of-care for all patients being evaluated for TB.

**Trial registration:**

US National Institutes of Health’s ClinicalTrials.gov, NCT03044158. Registered 06 February 2017.

Pan African Clinical Trials Registry, PACTR201610001763265. Registered 03 September 2016.

Contributions to the literature

- Trial design and execution may impact routine care procedures, making it harder to determine whether an intervention will improve patient- and public health-important outcomes when implemented in a real-world context.
- This paper describes the design and execution of an ultra-pragmatic trial to assess the effectiveness and implementation of a streamlined TB diagnostic strategy based around rapid, onsite molecular testing. Pragmatic design elements are emphasized.
- Trialists may find this paper a helpful roadmap to adopt a more pragmatic approach to study design and implementation in a global health context.

## Background

Early detection and treatment of tuberculosis (TB) are critical for reducing TB-related mortality and disease transmission. However, delayed and missed TB diagnoses remain pervasive in high-burden TB countries. Mathematical modeling suggests that an accessible rapid test with > 85% sensitivity and 97% specificity could avert between 12.3 and 22.4% of annual TB deaths, depending on rates of loss to follow-up [1]. Xpert MTB/RIF (Xpert) (Cepheid, Sunnyvale, CA, USA), a semi-automated, cartridge-based nucleic acid amplification test that can detect *Mycobacterium tuberculosis* and rifampicin resistance within 2 hours, is the first rapid TB test to approach these performance criteria. Since its endorsement as a replacement for smear microscopy by the World Health Organization in 2010, considerable global investment has been made to scale up Xpert testing capacity in high-burden countries. By the end of 2016, > 6500 GeneXpert modules and > 23 million Xpert cartridges had been procured by 130 countries with access to concessional pricing [2, 3]. However, the optimal strategy for implementing Xpert testing in high burden countries remains unknown.

High costs and infrastructure requirements restricted placement of the first generation of GeneXpert devices to higher-level facilities used by less than 15% of the population [4]. To increase access to Xpert testing, many high-burden countries have established specimen referral networks linking lower-level health facilities (*spokes*) to health facilities equipped with GeneXpert devices (*hubs*) [5–8]. However, studies have documented persistent challenges to the TB diagnostic evaluation process, including low utilization of Xpert, delays between sputum collection and return of Xpert results, and high rates of pre-treatment loss to follow-up for Xpert-positive patients [9–11].

The next generation of molecular diagnostics has been specifically designed for lower-level health centers, and thus has the potential to close these gaps in the TB diagnostic evaluation cascade of care. GeneXpert Edge is a battery-operated, single-module device equipped with cooling fans and dust filters and operated using a touchscreen tablet. Compared to the previous generation of GeneXpert devices, GeneXpert Edge is more durable, less costly, and designed to overcome limitations of resource-constrained settings, such as instability of the power supply and vulnerability to dust, heat, and humidity. These features have enhanced the potential of onsite Xpert testing as a viable alternative to sputum smear microscopy at lower-level health centers.

This paper describes the design of the GeneXpert Performance Evaluation for Linkage to Tuberculosis Care (XPEL-TB) trial, which seeks to evaluate streamlined TB diagnostic evaluation using onsite, rapid molecular TB testing. The scientific rationale for the proposed implementation strategy (referred to hereafter as “intervention strategy”) builds on work from a previous study by addressing key barriers to TB diagnostic evaluation at community health centers [12]. The aims of the XPEL-TB trial are to (1) compare the completion of TB diagnostic evaluation at community health centers (clusters) randomized to the intervention vs. routine TB diagnostic evaluation strategies, (2) identify processes and contextual factors that influence the implementation of the intervention strategy, and (3) compare the costs and epidemiological impact of the intervention vs. routine TB diagnostic evaluation strategies at all trial sites.

## Methods/design

### Study setting

The XPEL-TB trial is being conducted at 20 community health centers across the four administrative regions of Uganda to assess the effectiveness and implementation of the intervention in a mix of urban, peri-urban, and rural contexts. Uganda was selected as the trial setting for its high rates of TB and HIV/TB co-infection, with an estimated 86,000 TB cases and 34,000 HIV/TB cases in 2017 [13]. Uganda has been a regional leader in scaling up Xpert testing [2], and current Uganda National Tuberculosis and Leprosy Programme (NTLP) guidelines recommend Xpert as the first-line test for active pulmonary TB at health centers equipped with a GeneXpert device. At other health centers, including most community health centers, the guidelines recommend sputum smear microscopy as the initial test, followed by referral-based Xpert testing for smear-negative patients who have strong risk factors for TB (e.g., HIV infection) or a high clinical probability of TB after re-evaluation [14].

### Trial design

The XPEL-TB trial is an ultra-pragmatic, parallel cluster-randomized trial, with a hybrid type II effectiveness-implementation design [15]. An ultra-pragmatic approach, one in which all trial design elements included in the Pragmatic-Explanatory Continuum Indicator Summary 2 (PRECIS-2) tool are oriented toward evaluating the intervention strategy as it would be implemented in actual practice (i.e., towards the pragmatic end of the pragmatic-explanatory continuum), was selected to maximize applicability of results and to minimize interference with routine care [16]. The units of randomization and analysis (clusters) are community health centers that are part of Xpert referral networks in Uganda, with 10 sites per trial arm. Clusters rather than individual patients were randomized due to pragmatic feasibility, as well as concerns about contamination. Nested studies employing mixed-methods, health economics, and modeling methodologies are included in the trial in order to assess the implementation, cost-effectiveness, and epidemiological impact of the intervention strategy.

### Target setting and study sample

Site- and individual-level eligibility criteria are restricted to those that represent the real-world settings and patient populations receiving the intervention outside of a trial context. The ideal setting for onsite Xpert testing is the point of care nearest to the largest number of patients seeking care for TB symptoms (i.e., the lowest level of the health system); in Uganda, these are level III and level IV community health centers with TB microscopy units. Additional site-level inclusion criteria include the use of sputum smear microscopy as the primary method of TB diagnosis, participation in NTLP-sponsored external quality assurance for sputum smear microscopy, and linkage to a district or regional hospital for referral of samples for Xpert testing. For feasibility purposes, trial inclusion was also limited to health centers located within 150 miles of Kampala, as well as those that evaluated at least 150 patients for pulmonary TB and diagnosed a minimum of 15 smear-positive patients annually according to 2015 NTLP reporting data.

At the individual level, all adults (18+) who are evaluated for pulmonary TB at study sites (aim 1) during the trial period are included. Patients already diagnosed with TB (i.e., referred for treatment after being diagnosed elsewhere or already on TB treatment) or with a documented prior history of TB (due to concerns about false-positive Xpert results in this population) are excluded [17]. Surveys, direct observation studies, interviews, and/or focus group discussions (aims 2 and 3) are conducted in a subset of patients and providers involved in TB diagnosis and/or treatment at study sites.

### Informed consent

To ensure the trial captures complete data on all adults undergoing TB evaluation at study sites, a waiver of individual informed consent was obtained to extract patient-level data from Uganda NTLP TB registers used routinely by health facilities to record TB screening, testing, and treatment data. The waiver was justified on grounds that the intervention strategy poses no more than minimal risk to patients because it does not involve additional specimen collection and facilitates more rapid access to Xpert testing than would occur in routine care. However, informed consent is obtained from patients and providers who participate in surveys, direct observation studies, interviews, and/or focus group discussions related to the implementation and costing sub-studies.

### Intervention strategy

**Fig. 1:**
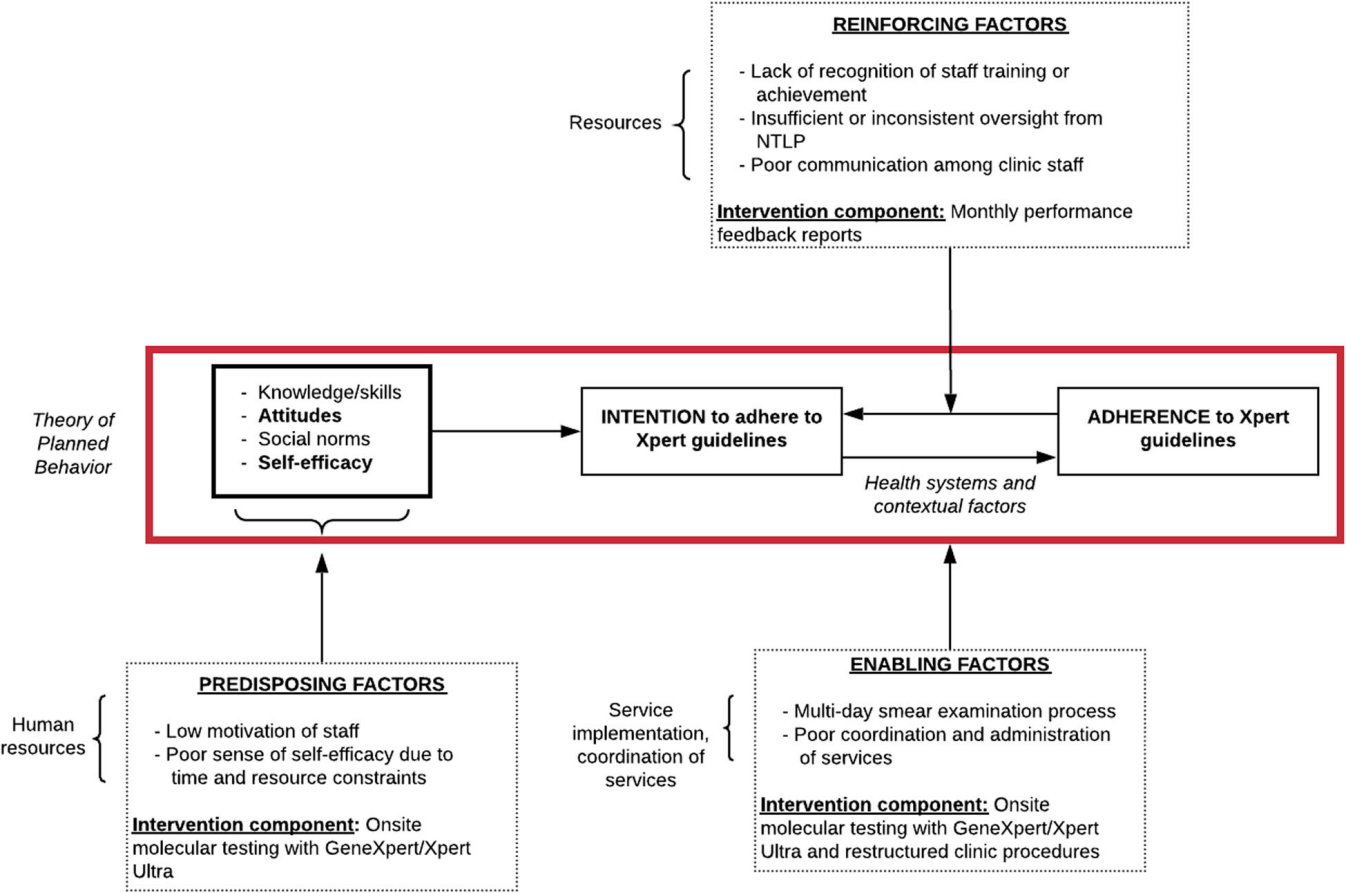
Theory-informed barrier assessment and design of intervention strategy

The theory-informed intervention strategy targets modifiable barriers of adherence to guidelines for TB diagnostic evaluation identified during formative work (Fig. 1) [12]. The Theory of Planned Behavior, which states that intention—mediated by knowledge, attitudes, subjective norms, and self-efficacy—predicts behavior (i.e., guideline adherence) [18] served as the conceptual model that informed the intervention strategy. The conceptual model also incorporated health system (financial and human resources, coordination of services, and service implementation) and patient (time and costs) factors likely to influence intention and adherence to TB evaluation guidelines. To inform the design of the intervention, identified barriers were prioritized in consultation with key stakeholders and then organized into three categories using the PRECEDE model: (1) *predisposing factors*—prior motives that either support or inhibit behavior, (2) *reinforcing factors*—rewards or punishments following a behavior or anticipated as a consequence of it, and (3) *enabling factors*—objective characteristics of an individual or environment that facilitate behavior [19]. The intervention strategy was developed to address each of these factors through the following components:

1. Onsite molecular testing using GeneXpert Edge: Patients presenting to health centers in the intervention strategy arm have access to rapid Xpert testing as a first-line TB test to target key predisposing (health worker time and workload constraints) and enabling (low sensitivity of sputum smear microscopy and inconsistent/delayed specimen transport to Xpert testing sites) factors. The trial began with intervention sites receiving a modified (external battery and dust filter) conventional one-module GeneXpert instrument (GeneXpert I), which was replaced with a GeneXpert Edge instrument once it became available in the third quarter of 2019.
2. Process re-design to facilitate same-day TB diagnosis and treatment: A standardized checklist was used to re-organize staff and patient workflow at intervention sites as an enabling factor to address the failure of patients to return after the initial health center visit. The process re-design focuses on ensuring that (1) TB screening was conducted at all clinic entry points using NTLP-mandated active case finding forms, (2) patients who screen positive were rapidly referred to the laboratory for sputum collection and testing, and (3) positive test results were reported immediately to patients and clinicians. Summaries of specific process changes made at each site are available in Additional file 1.
3. Performance feedback: Intervention strategy sites receive a monthly report card that includes site-specific TB diagnostic evaluation quality indicators as well as indicators averaged across all intervention strategy sites as a reinforcing factor to address the lack of communication between staff and limited oversight from district TB supervisors. Upon receipt of the report card, health center staff (along with the district TB supervisor when present) were required to identify barriers to improvement and develop plans to improve site performance following a Plan-Do-Study-Act model.

### Health center recruitment

A list of eligible health centers (i.e., clusters) that expressed interest in participating in the trial was reviewed with the Uganda NTLP and the National Tuberculosis Reference Laboratory (NTRL) Directors. Research staff met with the District Health Officer (DHO) for each approved site to inform him or her about the study and requested the participation of the site. If the request was granted, the DHO was asked to sign a Memorandum of Understanding describing key study procedures and expectations of participating sites. Study staff scheduled an enrollment visit with approved trial sites and followed a standard script to present the project to health center staff, including project aims and procedures. The final list of 20 sites to be randomized was selected from among sites deemed to be eligible, in consultation with the trial statistician to minimize potential for site-level variation that could impact power.

### Randomization

**Fig. 2:**
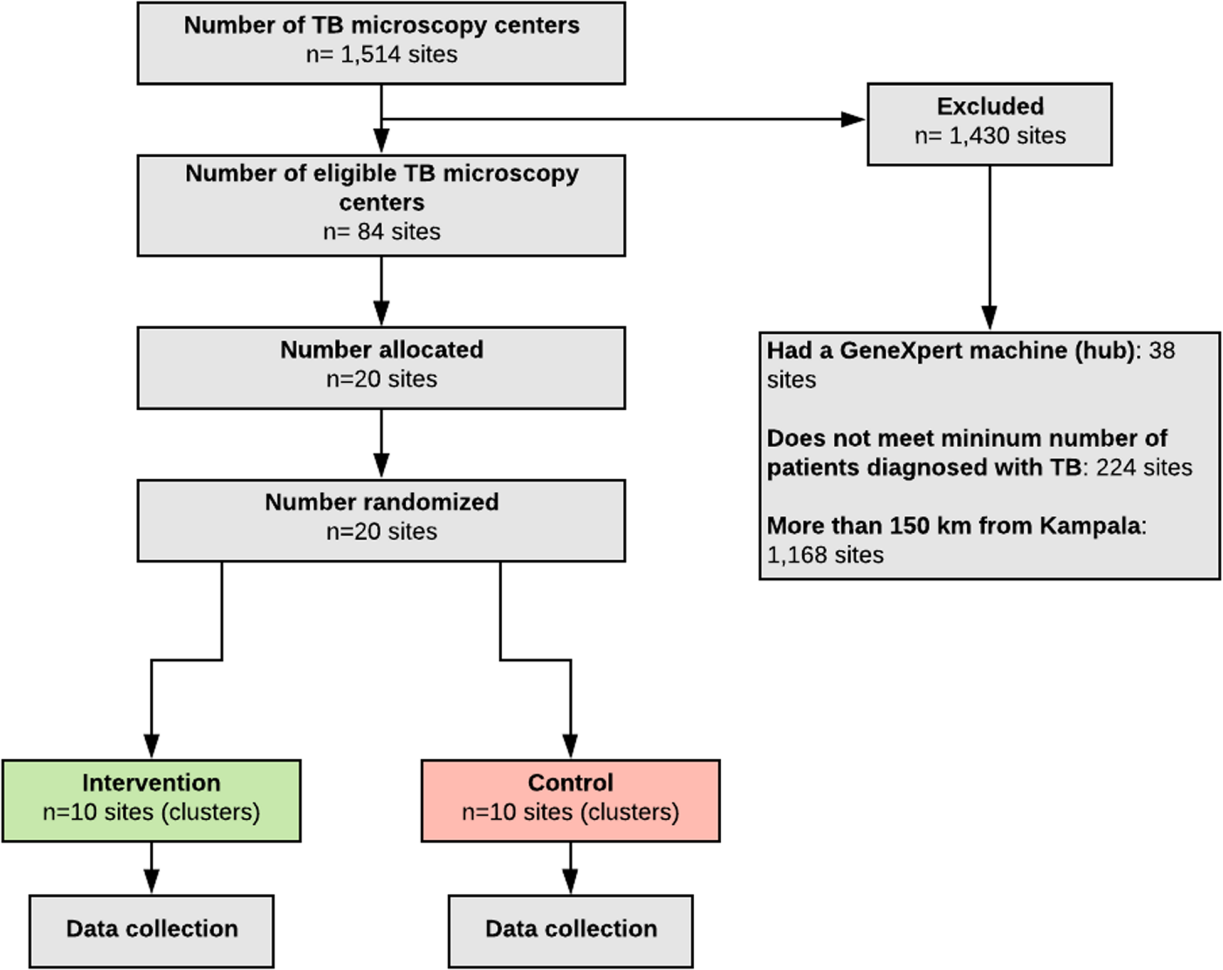
Site selection and enrollment for XPEL TB trial

Participating health centers were grouped into two equal-sized strata based on the proportion of patients with microbiologically confirmed TB initiated on treatment within 2 weeks of initial presentation at the health center in the year prior to the start of the trial. Stratification was used to help reduce between-cluster variation and enhance baseline balance with respect to the number of patients evaluated for and diagnosed with TB between arms. Site- and patient-level baseline characteristics strongly correlated with the proportion of microbiologically confirmed TB were identified; these included health center region, health center size, and HIV prevalence among TB patients. Restriction, a common approach in cluster-randomized trials with a small number of clusters, was used to help achieve baseline balance of these important site- and patient-level characteristics. The restriction factor was calculated, and the validity of the restriction was assessed using the validity matrix [20]. Random allocation sequences meeting stratification and restriction criteria were generated in STATA 14, resulting in 11,392 randomizations (StataCorp LP, College Station, TX). For convenience, 10,000 randomizations were randomly selected and each assigned a unique four-digit sequence (0000–9999). Health center representatives and district health officials were invited to participate in a randomization ceremony chaired by the Uganda NTLP Director. During the ceremony, four health center representatives were each requested to draw from a bag containing soccer balls numbered 0–9 to select the trial randomization sequence. The Uganda NTRL Director was then invited to select a final ball which determined the intervention strategy arm assignment based on being an odd or even number (Fig. 2).

### Blinding

Blinding of arm assignment is not feasible due to the nature of the intervention strategy. Investigators and study staff, with the exception of the trial statistician and data manager, are masked to ongoing aggregated patient-level data by study arm.

### Data collection and management

Patient-level data to assess processes of TB care are abstracted from routine data sources at trial sites and Xpert testing hubs (for control sites) for all eligible patients and entered into Research Electronic Data Capture (REDCap), a secure web-based application used for data collection and management in clinical research [21, 22]. Routine source documents include NTLP TB registers (presumptive, laboratory, and treatment registers), Xpert referral forms, and test data from GeneXpert devices. The procedures for patient-level data collection have been described in an earlier publication [5]. Briefly, health center staff not involved in TB diagnosis or treatment capture photos of source documents on password-protected smartphones and upload the photos to a secure central server every 2 weeks. Upon receipt, study staff review photos for completeness and accuracy, and attempts are made to address missing or conflicting data with health clinic staff. Study staff then abstract individual patient-level data on demographics, HIV and ART status, and TB testing from photos and enter the data into a password-protected REDCap study database. Data cleaning is performed regularly to identify missing or inconsistent information, and study staff resolve data queries during quarterly site visits through discussion with health center staff, review of primary data sources, and review of additional data sources (e.g., clinic registration book, ART register).

Patient-level data to assess health outcomes (vital status) are collected through a combination of patient phone calls and home visits. Phone calls and home visits are conducted between 6 and 8 months after patients initiate TB diagnostic evaluation.

Patient-level process and health outcome data collection is supplemented by focused data collection to assess implementation (fidelity and acceptability) (Table 1). Process metrics that reflect adoption of and fidelity to each component of the intervention strategy are assessed throughout the post-randomization period using test data downloaded quarterly from GeneXpert devices and performance feedback reports retrieved during quarterly site visits. Focus group discussions with health workers involved in TB diagnostic evaluation at each intervention strategy site are held at least 12 months after randomization to assess the acceptability of each intervention strategy component. Surveys with health workers (assessing theory of planned behavior constructs knowledge, attitudes, subjective norms, and self-efficacy related to TB diagnostic evaluation guidelines) and patients (assessing direct and indirect costs of and satisfaction with TB diagnostic evaluation) are conducted at all sites pre-randomization and at least 12 months after randomization to evaluate whether the intervention strategy modifies targeted barriers to TB diagnostic evaluation (see Fig. 1/Table 1). The surveys are conducted using validated patient cost and satisfaction with care questionnaires adapted to the local context [23, 24]. Last, in-depth interviews are conducted with health workers at high- and low-performing sites (based on analysis of patient-level, process metric, focus group, and survey data) after the trial is completed to better understand reasons for variability in adoption and implementation of the intervention strategy.

**Table 1:** Mixed methods evaluation strategy, sample, goal, data collection timing, and analysis

Strategy	Sample	Goal	Data collection	Analysis
Process metrics	All patients referred for TB diagnostic evaluation at intervention sites	Examine variability in adoption of and fidelity to intervention components across sites	Entire post-randomization period	Multivariate regression models
Surveys	1) Health workers involved in TB evaluation at all sites* 2) Sixty patients per site (20 patients/site, 400 total pre-randomization; 40 patients/site, 800 total post-randomization)**	Examine whether the XPEL TB strategy modifies targeted barriers to TB diagnostic evaluation	Once before and once after trial is completed	Multivariate regression models
Focus groups	Health workers involved in TB evaluation at intervention sites*	Assess acceptability of each component of the intervention strategy.	After trial is completed	Thematic interpretation
In-depth interviews	Purposive: 20–30 health workers at high- and low-performing intervention strategy sites (2–3/site)	Understand reasons for variability in adoption and implementation of the XPEL TB strategy	After trial is completed	Thematic interpretation

*Based on prior experience, we anticipate 5–10 staff members will be involved in TB diagnostic services at each health center (50–100 each at intervention and control health centers)

**Patients will be selected randomly within strata based on gender and timing of health center visit (undergoing TB testing vs. completed testing/initiating TB treatment)

Health economic data collection includes detailed budgetary analysis, interviews with key staff members, review of logbooks and/or timesheets to record proportions of staff time devoted to various activities, and time-and-motion studies of health workers involved in TB diagnostic evaluation.

### Trial outcomes

Trial outcomes were developed to address the five domains of the RE-AIM implementation research framework: depth of *reach* into a target population, *effectiveness* in closing gaps along the TB diagnostic evaluation cascade of care and improving health outcomes, factors that promote *adoption*, fidelity and costs of *implementation*, and factors that ensure *maintenance* over time (Table 2) [25]. The primary outcome reflects completion of the process of TB diagnostic evaluation and was originally the proportion of patients diagnosed with and treated for TB within 14 days. However, due to unexpected increases in the number of patients being tested for TB at intervention sites during the first 6 months of the trial, the Trial Steering Committee approved changing the primary outcome from a proportion to the absolute number diagnosed with and treated for TB within 14 days. Details on the rationale for and implications of changing the primary outcome from a proportion to a count are described in Additional file 2. Additional outcomes by RE-AIM domain are presented in Table 2.

**Table 2:** Trial outcomes categorized using the RE-AIM framework

RE-AIM domains	Trial outcomes
Reach	• Number/proportion completing Xpert testing
Effectiveness	Process outcomes • Number/proportion diagnosed with and treated for TB within 14 days • Number/proportion diagnosed with microbiologically-confirmed TB • Time to diagnosis of microbiologically-confirmed TB • Number/proportion diagnosed with rifampin resistance • Number/proportion with microbiologically-confirmed TB completing treatment Health and health economic outcomes • Number/proportion who died within 6 months • Number/proportion with microbiologically-confirmed TB who died within 6 months • Number/proportion treated for TB who died within 6 months • Incremental cost-effectiveness ratio
Adoption	• Proportion of eligible sites able and willing to initiate each component of the intervention strategy
Implementation	Fidelity • Process metrics for each intervention strategy component • Proportion tested by Xpert • Number of GeneXpert device non-operation days • Proportion of invalid, error, or indeterminate Xpert results • Proportion treated on same-day if Xpert-positive • Proportion of performance feedback report cards reviewed at staff meetings • Qualitative data from health worker focus group discussions and in-depth interviews Acceptability • Thematic output from health worker focus group discussions Patient and health system costs • Incremental cost of introducing and maintaining the intervention strategy • Incremental patient cost per diagnostic evaluation and per treatment initiated Modification of targeted barriers • Median scores for health worker knowledge, attitudes, subjective norms, and self-efficacy related to TB diagnostic evaluation guidelines • Thematic output from provider in-depth interviews • Median patient costs associated with completing TB diagnostic evaluation • Median scores for domains of patient satisfaction questionnaire

### Sample size and power considerations

The number of trial sites was chosen based on feasibility considerations. The number of participants and resulting trial duration were chosen using the proportion of patients diagnosed with and treated for TB within 14 days as the primary outcome. Sample size calculations appropriate for cluster-randomized trials used the following assumptions: 10 clinics per trial arm, an 18-month trial duration, a type I error of 5%, and a power of 90% [20]. Pre-randomization data collected from the 20 trial sites between January and December 2017 showed that 6.7% of patients referred for TB evaluation were initiated on treatment for confirmed TB within 2 weeks, with a 0.36 coefficient of variation (*k*) between clusters and a harmonic mean of 268 eligible patients enrolled at each health center (cluster). Based on these conditions and allowing for 10% missing data, an 18-month trial duration was chosen to achieve a target sample size of 5360 patients. Power and detectable effect size calculations were repeated when the primary outcome was approved for change from a proportion to a count. Using the same trial parameters (10 clinics per trial arm, 18-month trial duration, and a mean of 268 patients per cluster) and assuming a 0.2–0.3 within-arm standard deviation of the log-transformed outcome based on pre-trial data, the trial has 80–90% power to detect a geometric mean ratio in the number of TB cases diagnosed and treated within 14 days of 1.30–1.58, comparing the intervention and control arms. This range of detectable effect sizes is reasonable given that Xpert MTB/RIF is twice as sensitive as smear microscopy (i.e., double the number of confirmed TB cases), and onsite testing is expected to reduce pre-treatment loss to follow-up by at least half (from 30 to < 15%).

The sample size for nested studies is either fixed by parameters of the clinical trial (process metrics, provider surveys, and provider focus groups) or based on feasibility considerations (patient surveys and provider in-depth interviews). For quantitative analyses, the sample size is sufficiently large (data on approximately 11,283 patients and 180 performance feedback reports for process metrics, 800 patient surveys, and 100–200 provider surveys) to enable multivariable analysis to identify factors associated with adoption and implementation of the intervention strategy.

## Analysis

### Analysis of the primary outcome

The primary analysis will be conducted at the cluster (clinic)-level. Poisson regression or negative binomial regression will be used, depending on whether there is evidence for over-dispersion. An offset at the clinic level representing days contributing patients to the study will be applied if any trial site unexpectedly withdraws from the study. The model will adjust for randomization strata and the count of microbiologically confirmed TB cases initiated on treatment within 14 days in the 12-month period before the trial start date.

### Analysis of implementation outcomes

A cluster-level analysis will be performed to compare the change from baseline (pre-randomization) to post-intervention in patient cost, patient satisfaction with care, and health worker beliefs about TB diagnostic evaluation guidelines. Identification of patient-, provider-, and/or clinic-level factors associated with the adoption and implementation of intervention components (as determined by process metrics) will be done through linear or logistic regression.

Qualitative analyses will involve collaborative development of a coding framework and detailed coding of transcripts using the qualitative software analysis program Dedoose [26]. Coded transcripts will be sorted to identify thematic groupings. Thematic interpretation will focus on the individual, social, and structural factors associated with successful or unsuccessful adoption and/or implementation of the intervention components at different sites.

### Trial rollout

After enrollment into the study, a visit was made to each trial site and affiliated Xpert testing hub (for control sites) to assess the completeness of TB register data and conduct refresher trainings on Uganda NTLP guidelines (trial sites) or GeneXpert device maintenance and operation (Xpert testing hubs). At each site, baseline data were collected for the 12-month period prior to the planned trial start date to facilitate stratified and restricted randomization.

In the 3 months prior to the planned trial start date, a 2-day site visit was made to each trial site. At control sites, key messages related to TB guidelines were re-emphasized, including processes for referral of sputum samples for Xpert testing. Laboratory staff received refresher training on sputum collection and sputum smear microscopy, including proficiency testing using panel slides. Laboratory and drug inventories were reviewed to ensure adequate supply of sputum collection cups, glass slides, staining reagents, and TB drugs. Completeness of TB laboratory and treatment registers was assessed, and re-training provided as needed. Guidelines refresher and TB register completion trainings were also provided at the intervention sites. Additionally, at intervention sites, a GeneXpert I device, battery, and solar panel were installed, and laboratory staff were trained on sputum collection, Xpert testing, documentation, and device maintenance. Laboratory and clinical staff were trained on interpretation of Xpert results. Health center staff were engaged in a discussion about laboratory, clinical, and pharmacy workflow reorientation, with an emphasis on prompt identification and referral of patients with possible active TB for sputum collection, onsite molecular testing, communication of diagnostic results to clinicians, and same-day treatment initiation for Xpert-positive patients. Finally, all staff were introduced to performance feedback report cards and given expectations for review and follow-up actions.

Research staff visit all trial sites quarterly to resolve missing data queries, collect health system cost data, and conduct provider surveys and focus group discussions. In addition, a report card is generated for intervention sites for distribution each month. Upon receipt of the report card, sites complete an attached worksheet to describe any barriers to performance, as well as goals to improve performance. No further follow-up is done with these report cards and/or worksheets. All other contact between research staff and health center staff is kept minimal.

## Discussion

Novel TB diagnostic tests such as onsite Xpert testing have the potential to minimize attrition along the TB care cascade and thereby improve rates of TB diagnosis and treatment. Evaluation of these tests and associated implementation strategies should be conducted within settings and populations of intended use to demonstrate their expected impact after scale-up. However, trial design and execution features—even for those labeled as pragmatic—have often utilized resources or procedures that are not available in routine care and exclude key patient sub-populations [27]. These design choices can reduce attrition along the TB diagnostic cascade of care in both trial arms, affecting the ability of a trial to demonstrate the impact of novel TB diagnostic tests and strategies relative to what might occur in usual care.

The XPEL-TB trial combines an ultra-pragmatic approach to implementation with a hybrid type II design to (1) demonstrate the expected impact of onsite Xpert testing on patient-important outcomes and (2) explain why and how the intervention strategy based around onsite Xpert testing did or did not work. The ultra-pragmatic approach minimizes the impact of trial procedures on routine care practice, limiting the potential impact on key primary and secondary outcomes. This is particularly noteworthy in the context of previous TB diagnostic studies, which were either implemented in specialized settings (e.g., tertiary health centers) or used resources and/or procedures not available in routine care such as on-site research staff, additional diagnostic testing (chest X-ray and culture), or financial incentives to minimize patient loss to follow-up [28–30]. Additionally, narrowly defined inclusion criteria as commonly seen in many TB diagnostic trials can restrict enrollment to patients with a higher pre-test probability of TB or patients more likely to comply with follow-up. These features are likely to improve outcomes in both trial arms, making it difficult to assess the true impact of the intervention. Thus, each design element of the XPEL-TB trial was oriented towards the ultra-pragmatic end (score of 5) of the pragmatic-explanatory continuum, per scores generated using the PRECIS-2 tool (Table 3) [16]. To the best of our knowledge, this is among the first TB diagnostic trials that does not involve formal recruitment and consent procedures and uses data abstracted from routine data sources to assess the primary and key secondary outcomes.

**Table 3:** XPEL-TB PRECIS-2 domains

PRECIS-2 domain	Assessment of XPEL-TB	Rating (1–5, where 5 is very pragmatic)
Eligibility	Nearly all adults who would have been offered Xpert testing if available in routine care are included in the trial. Only patients with a previous history of TB are excluded (to not falsely increase the primary outcome).	5
Recruitment	No formal recruitment procedures are used. A waiver of consent was obtained to enable automatic inclusion of data on all adults undergoing TB evaluation at trial sites. No incentives given to patients in either arm.	5
Setting	The trial is implemented at 20 sites at the lowest level of the health system where TB diagnostic services (sputum smear microscopy) are provided—the sites targeted for expansion of Xpert testing using next-generation platforms.	5
Organization	Xpert testing is implemented using existing healthcare staff and infrastructure at trial sites.	5
Flexibility: delivery	Process re-design and performance feedback were adapted by each trial site to suit its needs and processes of care and supervision.	5
Flexibility: adherence	No additional incentives or procedures are in place to encourage patients in the intervention arm with microbiologically-confirmed TB to initiate on treatment.	5
Follow-up	Process and outcome data are collected from routine clinic records. No onsite research staff to conduct patient enrollment, data collection, or follow-up, with the exception of vital status assessment.	5
Primary outcome	The primary outcome is relevant to TB patients, their relatives, clinic staff, and the Uganda NTLP	5
Primary analysis	No special allowances will be made in the primary analysis for non-adherence or variability in implementation by site	5

A hybrid type II effectiveness-implementation design was selected to facilitate concurrent evaluation of the clinical effectiveness and implementation of onsite Xpert testing. The dual focus on effectiveness and implementation is innovative in global health diagnostics. Previous trials of Xpert testing have not included co-interventions to support implementation and were designed primarily to evaluate effectiveness, with minimal attention given to why and how Xpert testing did or did not work [31]. Such information is critical to guide scale-up efforts in Uganda and in other settings.

It is expected our trial design and implementation approach will influence our findings, as well as inform next steps for clinical practice and research. We anticipate that implementation under real-world conditions will help demonstrate an increase in the number of patients diagnosed and treated for active pulmonary TB within 2 weeks of initial presentation at the health center. If indeed the case, this finding would diverge from most published data on the effectiveness of Xpert testing on patient-important outcomes, such as mortality, and could result in more support and funding for GeneXpert scale-up worldwide. In addition, qualitative data on fidelity of implementation, barriers to uptake, and variability of uptake as well as data on cost and cost-effectiveness will inform modifications to intervention components beyond the conclusion of the trial.

## Supplementary information

**Additional file 1.** Summaries of specific process changes made at each site.

**Additional file :.** Details on the rationale for and implications of changing the primary outcome from a proportion to a count.

## Data Availability

Not applicable

## Abbreviations

DHO: District Health Officer
FGD(s): Focus group discussion(s)
NTLP: National Tuberculosis and Leprosy Programme
NTRL: National Tuberculosis Reference Laboratory
PRECIS-2: Pragmatic-Explanatory Continuum Indicator Summary 2
REAIM: Reach, effectiveness, adoption, implementation, maintenance
REDCap: Research Electronic Data Capture
TB: Tuberculosis
XPEL-TB: Xpert Performance Evaluation for Linkage to Tuberculosis Care
Xpert: Xpert MTB/RIF
