# Supplementary material for: Study protocol: a cluster randomized trial to evaluate the effectiveness and implementation of onsite GeneXpert testing at community health centers in Uganda (XPEL-TB)
Source: Implement Sci. 2020 Apr 21;15:24. doi: 10.1186/s13012-020-00988-y (PMC7171793; doi:10.1186/s13012-020-00988-y)
Supplement: Supplementary file 1 — Additional file 1. Summaries of specific process changes made at each site. [file 13012_2020_988_MOESM1_ESM.docx]

***Additional file 1***: **Process re-design at intervention sites**

| **Intervention Sites** |  | 1. TB screening using a standardized intensified TB case finding form | 2. TB screening at all entry points | 3. Patients with TB symptoms immediately referred to lab for sputum submission | 4. On-demand testing of sputum samples | 5. Patients with confirmed TB escorted to clinician for treatment initiation |
| --- | --- | --- | --- | --- | --- | --- |
|  | 1 | x | x | x | x |  |
|  | 2 |  | x | x | x |  |
|  | 3 | x | x | x | x |  |
|  | 4 | x | x | x | x | x |
|  | 5 | x |  |  |  | x |
|  | 6 |  | x | x | x |  |
|  | 7 |  | x | x | x |  |
|  | 8 |  | x | x | x | x |
|  | 9 |  | x | x |  |  |
|  | 10 |  | x | x | x | x |

Details on process re-design activities

1. An intensified TB case finding form issued by the Ministry of Health in Uganda is used to screen patients for TB symptoms. The form prompts health workers to screen patients for persistent cough, fever, weight loss, or night sweats, and recommends all patients with one or more of these symptoms be evaluated for TB.
2. Patients are screened for TB at all entry points where patients register to be seen for care. Patients at site 5 are screened for TB at select entry points only.
3. Patients identified as having TB symptoms following screening are immediately sent to the laboratory for sputum submission instead of first waiting to see a clinician.
4. Sputum samples are tested immediately following collection instead of being batched for testing at select times during the day.
5. Patients are asked to wait for Xpert results and are escorted by laboratory staff to a clinician for treatment initiation if results are positive.
