# Supplementary material for: Study protocol: a cluster randomized trial to evaluate the effectiveness and implementation of onsite GeneXpert testing at community health centers in Uganda (XPEL-TB)
Source: Implement Sci. 2020 Apr 21;15:24. doi: 10.1186/s13012-020-00988-y (PMC7171793; doi:10.1186/s13012-020-00988-y)
Supplement: Supplementary file 2 — Additional file :. Details on the rationale for and implications of changing the primary outcome from a proportion to a count. [file 13012_2020_988_MOESM2_ESM.docx]

***Additional file 2: Change in Primary Outcome for XPEL TB Trial***

The primary outcome for the trial was officially changed in May 2019, approximately 6 months into the 18-month trial, following approval by the Trial Steering Committee (TSC). Below, we present the rationale for changing the primary outcome as approved by the TSC:

**Original primary outcome:** Proportion treated for microbiologically-confirmed TB within two weeks of referral for sputum-based TB testing

**New primary outcome:**  Number treated for microbiologically-confirmed TB within two weeks of referral for sputum-based TB testing

**Rationale for new primary outcome:** The new primary outcome better reflects the intended effect of the multi-component intervention to drive more people who present to health facilities through the entire TB diagnostic evaluation cascade of care, including being screened for TB, tested for TB (if screen-positive), diagnosed with TB (if tested) and initiated on treatment (if diagnosed). Process re-design is expected to help improve the number screened and tested. Onsite molecular testing is expected to increase the numbers diagnosed (increased sensitivity) and treated (faster turn-around time and reduced workload). Performance feedback is expected to maintain improvement along each step of the cascade. Thus, the intervention is expected to increase both the number of patients entering and completing the cascade of care.

Since the trial started, the number referred for TB testing has been significantly higher than what was expected based on pre-trial data at intervention health centers. As more patients enter the cascade of care and are referred for TB testing, the proportion diagnosed and treated is decreasing (3% across arms since trial started vs. 6% across arms in pre-trial period) due to a greater increase in the number referred for testing (denominator) relative to the number diagnosed and treated (numerator). The absolute number diagnosed and treated for TB therefore better reflects whether the intervention is working as intended. A similar count outcome was used for the same reasons as the primary outcome for a recently-published trial of active case finding in South Africa:

<https://journals.plos.org/plosmedicine/article?id=10.1371/journal.pmed.1002796>

**Experience with changing primary outcome based on the literature**:

- Changing the primary outcome is fairly common - In a systematic review, Ramagopalan et al found almost a third of trials did so (<https://www.ncbi.nlm.nih.gov/pmc/articles/PMC4032105/>)
- Our decision to change the primary outcome follows best practice recommendations on how/when this should be done (<https://www.ncbi.nlm.nih.gov/pmc/articles/PMC1852589/>).
  - Assumptions made during the design of the trial were not accurate (*i.e.,* testing volume would remain the same as in the two-year baseline period)
  - Decision to change the primary outcome was based on data available in trial enrollment reports (without any interim analysis of the original primary outcome)
  - External advisory board consulted and approved the change (meeting date: 5/17/2019)

**Revised detectable effect size calculations for new primary outcome**

We first used 10 months of pre-intervention period data across all 20 clinics to assess 1) the geometric mean number of patients diagnosed and treated for TB within 14 days (new primary outcome), the natural log (ln) of the geometric mean and 3) the standard deviation of the natural log at control sites, intervention sites and overall.. For the pre-intervention period, the intervention clinics have a *lower* geometric mean number of clinic attendees who were diagnosed with TB and started on TB treatment within 14 days compared with the control arm:

|  | **Geometric mean** | **Mean of ln outcome** | **SD of ln outcome** |
| --- | --- | --- | --- |
| Control | 14.70 | 2.69 | 0.23 |
| Intervention | 6.49 | 1.87 | 0.32 |

We then estimated the detectable effect size (expressed as a geometric mean ratio). The calculations assume the same parameters as for the original primary outcome (10 clinics/arm, 18-month trial duration to achieve geometric mean of 286 patients/cluster in control and intervention arms). Table 1 below shows the detectable effect size assuming within-arm SD of ln outcome of 0.2 to 0.3 and power of 80-90%. The detectable effect sizes shown below are conservative (calculations do not take into account the baseline differences between arms in the new primary outcome and are based on 10-months rather than full 24-months of pre-intervention data). Final calculations will be repeated once baseline data is fully entered and available for analysis.

**Table 1: Detectable effect sizes**

| **SD of ln outcome** | **Geometric Mean Ratio of** **the new primary outcome**  **(intervention vs control)** | |
| --- | --- | --- |
|  | **80% power** | **90% power** |
| 0.2 | 1.30 | 1.36 |
| 0.3 | 1.49 | 1.58 |

We believe a GMR of 1.30-1.58 is a reasonable detectable effect size. Xpert MTB/RIF is twice as sensitive as smear microscopy (double the number of confirmed TB cases) and we expect onsite testing to reduce pre-treatment loss to follow-up by at least half (from 30% to <15%).

Analysis:

The primary analysis will be conducted using a poisson model, with an offset at the clinic level of the total number of days the clinic contributes individuals to the study (starting from 22 October 2018). Evidence for over-dispersion will be assessed and if over-dispersion is detected then negative binomial regression will be used, using the same offset. Either model will adjust for randomization strata, as well as the pre-intervention period data on the number of patients diagnosed with TB and started on treatment within 14 days. The exact functional form of this covariate will be identified through fractional polynomials. Further adjustment for clinic-level factors that show imbalance will be explored, though the number of adjustment factors will be limited as all are at the clinic-level. Adjusting for baseline differences is preferred to the approach of comparing the change from baseline in the two arms as it results in an unbiased estimate of the intervention effect and with a gain in precision for the intervention effect.
